# Transcriptome and Metabolome Insights into Key Genes Regulating Fat Deposition and Meat Quality in Pig Breeds

**DOI:** 10.3390/ani14243560

**Published:** 2024-12-10

**Authors:** Suthar Teerath Kumar, Yunlong Zheng, Jing Xu, Ziyi Zhao, Qi Zhang, Yunpeng Zhang, Min Li, Hong Zou, Riaz Muhammad Azeem, Wu-Sheng Sun, Yuan Zhao, Shu-Min Zhang

**Affiliations:** 1 Key Laboratory of Animal Production, Product Quality and Security, Ministry of Education, College of Animal Science and Technology, Jilin Agricultural University, Changchun 130118, China; teerathkumarsuthar@gmail.com (S.T.K.); z15845337936@163.com (Y.Z.); 13689759057@163.com (J.X.); 18031975634@163.com (Z.Z.); zhangyunpeng1218@163.com (Y.Z.); 15931321811@163.com (M.L.); 2 Institute of Animal and Veterinary Sciences, Jilin Academy of Agricultural Sciences, Changchun 130033, China; zhang12100214@163.com; 3 Jilin Provincial Engineering Research Center of Animal Probiotics, Jilin Provincial Key Laboratory of Animal Microecology and Healthy Breeding, Engineering Research Center of Microecological Vaccines (Drugs) for Major Animal Diseases, Ministry of Education, College of Animal Science and Technology, Jilin Agricultural University, 2888 Xincheng Street, Changchun 130118, China; b18204370678y@163.com (H.Z.); sunwsh@jlau.edu.cn (W.-S.S.); 4 Jilin Provincial Engineering Research Center of Animal Probiotics, Jilin Provincial Key Laboratory of Animal Microbiological Vaccine (Durg) for Major Animal Diseases, Ministry of Education, Collage of Veterinary Medicine, Jilin Agricultural University, Changchun 130118, China; azeem.riaz786@outlook.com

**Keywords:** commercial LWLDP breed, insulin signaling pathway, muscle metabolomics, meat quality traits, Songliao Black Pig, transcriptomics

## Abstract

Meat quality is a complex trait that exhibits significant variation across pig breeds, and the regulatory mechanisms governing pork meat quality are not fully elucidated. We compared the transcriptomics and metabolomics of the longissimus dorsi (LD) muscle between the Songliao Black Pig (SBP) and Large White × Landrace Pig (LWLDP) to investigate breed-specific differences in meat quality and underlying regulatory pathways. The results showed that SBP meat had a higher marbling score and backfat thickness, a richer color, a lower shear force, and reduced drip loss. Fatty acid (FA) analysis identified 15 significant FAs in the LWLDP, with docosahexaenoic acid (DHA) in the SBP, while amino acid (AA) analysis revealed no breed-based differences. Transcriptome analysis identified 134 upregulated and 362 downregulated genes in the SBP. Protein–protein interaction (PPI) network analysis found 25 key genes, which are associated with muscle development, fat deposition, and overall meat quality, while genes in the insulin signaling pathway, such as PPP1R3B, PPARGC1A, SOCS1, EIF4E, PRKAR2A, PRKAG2, and FASN, play a crucial role in balancing fat metabolism and catabolism. Metabolomic analysis identified 89 upregulated and 10 downregulated metabolites in the SBP, primarily involved in fructose and mannose metabolism, amino acid biosynthesis, nucleotide sugar metabolism, and glucagon signaling pathways. Gene–metabolite association analysis found that the PPP1R3B gene had a strong association with Thr-Leu, Maltol, D-myo-Inositol-4-phosphate, and Fructose-6-phosphate, while MYOG correlated with Mannose-6-phosphate, Fructose-1-phosphate, Mannose-1-phosphate, and Glucose-6-phosphate. In contrast, NR4A3 and PPARGC1A showed a strong negative correlation with most upregulated metabolites. In conclusion, this study identified functional genes, elucidated the mechanisms associated with meat quality traits, and identified gene–metabolite associations involved in energy metabolism, muscle development, and fat deposition, providing valuable insights into the molecular mechanisms that regulate meat quality between pig breeds.

## Simple Summary

Understanding the factors that affect pork meat quality is essential for improving meat production and consumer satisfaction. Meat quality, such as tenderness, flavor, and color, is influenced by various biological processes in pigs, including the fat content and how it is distributed. This study compared two pig breeds—the Songliao Black Pig (SBP) and a commercial crossbreed of Large White × Landrace (LWLDP)—to uncover the genetic and metabolic factors that influence these qualities. We found that the SBP generally had a superior meat quality, with higher marbling (fat within the muscle), richer color, better texture, and less moisture loss compared to the crossbreed. By analyzing fats, amino acids, and genes, we identified key genes and metabolic pathways that regulate fat deposition and overall meat quality. This research helps identify genetic markers and biochemical processes that could be targeted to enhance meat quality in pork production, supporting the breeding of pigs that meet consumer demands for taste, texture, and nutritional value.

## 1. Introduction

Pork is the most widely produced and consumed meat globally and plays a significant part in meat consumption. People prefer high-quality meat as their level of living rises, seeking to improve the sustainability issues concerning the production and consumption of meat [1]. Meat quality directly affects human health and life, in addition to being a crucial economic characteristic for pork enterprises [2]. Meat quality is a broad term that is challenging to quantify straightforwardly and uniquely. Consumers choose pork based on factors such as color, marbling, pH, tenderness, and water loss when evaluating meat quality [3]. The degree of marbling in meat is a measure of the equilibrium between the synthesis, breakdown, and absorption of triglycerides. Although they primarily remain in adipocytes, triglycerides can also be found in the cytoplasm of muscle fibers, where they are present as droplets [4]. The sensory acceptance, water-holding capacity, and softness of pork are strongly connected with the marbling score; pork’s flavor and juicy texture are improved when the marbling score/IMF content exceeds 2.5 percent [5,6,7]. Pork’s potential eating quality can be determined by looking at its marbling score and muscle fiber type, which are linked to a specific flavor, juiciness, and tenderness [8,9]. Another crucial component of meat quality is its nutritional composition, e.g., FAs, which are some of the main components influencing the taste and softness of meat since they are precursors to fat. In addition, a variety of AAs, including leucine, lysine, glutamate, and aspartic acid, significantly affect flavor, and some of them are good markers of meat’s protein content [10]. Mass spectrometry (MS) has become a popular technique for identifying and measuring a wide range of chemicals in different muscle samples [11]. Meat-based metabolites have also been demonstrated to distinguish between live and dead pork [12], as well as different pig breeds [13]. These metabolites can be used to distinguish between different breeds of pigs and are useful in understanding the chemical makeup of meat quality.

Many factors, such as environmental factors, nutritional management, age, sex, and genetic factors, affect the quality of meat [14]. Several Western pig breeds, such as Duroc-Yorkshire–Landrace (DYL), Puławska, Polish Large White, Pietrain Pigs, and the LWLDP, among others, hold the highest market share in China and globally due to their quick growth rate, high feed conversion ratio, and apparent economic benefits due to their stable genetics and suitability for crossbreeding and lean meat production. However, previous studies showed that native pig breeds have a higher meat quality than commercial breeds [15,16,17,18]. Different studies have been carried out to identify differentially expressed genes (DEGs) linked to meat quality by utilizing transcriptome data from the adipose tissues and longissimus dorsi muscles of Chinese indigenous pig breeds and Western commercial pig breeds [19,20,21]. Previous research has focused on identifying quantitative trait loci (QTLs) and intrinsic genes related to meat quality and examining how these genetic factors influence meat quality traits at the molecular level [22,23,24]. Additionally, a number of genes are linked to the quality of pig meat, including isocitrate dehydrogenase (NADP(+)) 2 (IDH2), succinate-CoA ligase GDP-forming subunit beta (SUCLG2), ELOVL fatty acid elongase 5 (ELOVL5), and myogenic differentiation 1 (MYOD1) [19,25,26]. However, previous research has focused on several Chinese indigenous pig breeds, but data about the extent of variation in meat quality attributes are still insufficient and they are unable to effectively depict the distinct variety of meat quality across Eurasian pig breeds.

The Songliao Black Pig (SBP) is the first ever lean meat Black female line breed of the northeastern regions of China, adapted to the high cold environment (−28 °C) in winter [27]. The SBP has been well-known for its higher marbling score, backfat thickness, lean meat, tender texture, delicious taste, and rich flavor [28]. There are notable differences in marbling, backfat thickness, and other meat quality traits between the SBP and Western pig breeds. However, the mechanisms underlying these differences remain unclear. Therefore, the SBP could be an ideal animal model for investigating the fat deposition and the regulatory mechanisms involved in developing superior meat quality traits. This study aims to compare the LD muscle in terms of meat quality traits, FA profiles, AA profiles, transcriptomics, and muscle metabolomics profile between the SBP and Large White × Landrace Pig (LWLDP) breed and insights into molecular mechanism influencing fat deposition and metabolic pathways that contribute to desirable meat quality. These insights may aid in the development of new strategies to produce high-quality pork.

## 2. Materials and Methods

### 2.1. Ethics Statement

All animal procedures were applied as per guidelines approved by the Institutional Animal Care and Use Committee (IACUC) of the Jilin Agricultural University. The study protocol was approved by the IACUC under approval number (KT2023023).

### 2.2. Animal Housing, Sex, and Feeding Management

We reared the one hundred male pigs (fifty Songliao Black Pigs (SBP) and fifty Large White × Landrace Pig (LWLDP) breeds) at Gongzhuling National Agricultural Science and Technology, Feimas Animal Husbandry Co., Ltd., Gongzhuling, China for meat quality analysis. All pigs were raised under pen housing with uniform conditions, including consistent temperature, humidity, and ventilation; ad libitum access of same diet was provided at 6:00 a.m. and 12:00 p.m. daily throughout their entire lifespan (Table S1). Water was provided through nipple drinkers all the time.

### 2.3. Slaughtering Age, Method, and Sample Collection

All pigs with an average age of 210 ± 15 days were transported to Gongzhuling Gaojin Food Co., Ltd, Gongzhuling, China for slaughtering. Initially, we measured the live weight (105.40 ± 4.3 kg for SBP and 121.23 ± 5 kg for LWLDP) of all pigs at slaughterhouse. Then, after, animals were humanely euthanized using electric shock followed by rapid exsanguination. Further, samples were collected from the longissimus dorsi (LD) muscle, placed in a plastic zip bag, and immediately stored in ice box for meat quality assessment. Additionally, a small portion of LD muscle was wrapped in aluminum foil, rapidly snap-frozen in liquid nitrogen and stored at −80 °C for subsequent analysis. Furthermore, we assessed the meat quality traits including marbling score, backfat thickness, meat color, drip loss, shear force, and pH24 of all pigs to evaluate the difference in meat quality between breeds. After this, five pig samples from each breed were randomly selected for further fatty acids, amino acids, and transcriptome and metabolome analysis.

### 2.4. Meat Quality Assessment

The samples of LD muscle were collected at the region of 7th and 8th vertebrae on the left side of the carcass within 30 min post slaughter for evaluating the meat quality. We removed the visible intermuscular fat from the samples. Meat quality was assessed using the guidelines suggested by the Ministry of Agriculture and Rural Affairs of the People’s Republic of China (NY/T 821-2004; NY/T 1180-2006). Backfat thickness was measured using a vernier caliper at the 7th vertebra, in accordance with (NY/T 2894-2016). The pH values were recorded 24 h after slaughter using a portable pH meter (PHS-3DW, Beijing Shunkeda Technology, Beijing, China) in triplicate. Meat color and marbling were evaluated separately according to the National Pork Producers Council (NPPC, 2000) scoring system, with color scores ranging from 1 (pale) to 5 (dark) and marbling scores from 1 (devoid) to 5 (abundant), allowing for 0.5 increments between scores. Drip loss was measured by cutting a sample from the LD muscle between the seventh and eighth thoracic vertebrae, recording its initial weight, suspending it in an inflated bag to prevent contact, and storing it at 4 °C for 48 h. After this, sample was gently blotted dry and weighed, with measurements taken in quadruplicate. For shear force measurement, LD muscle samples were first cooled to 4 °C, then cooked to an internal temperature of 80 °C in a boiling water bath, and cooled back to 4 °C. Three cylindrical cores with 1.27 cm in diameter were cut from each cooked steak parallel to the muscle fibers. The shear force was measured using a digital tenderness testing machine (C-LM4, Northeast Agricultural University, Harbin China) by shearing each sample perpendicular to the long axis of the core.

### 2.5. Fatty Acid and Amino Acid Content Analysis

The fatty acid (FA) composition of intramuscular fat (IMF) was analyzed using a gas chromatograph (Shimadzu GCMS-TQ8040 NX Triple Quadrupole) using the method suggested [29]. Amino acid (AA) was determined through an ion-exchange amino acid analyzer (L8900, Hitachi, Tokyo, Japan), with the method suggested in [30].

### 2.6. Library Construction and Sequencing

Five biological replicates per breed with two replications of each sample were used for sequencing. Total RNA was extracted from LD muscle tissue using Trizol Reagent (Invitrogen Life Technologies, Carlsbad, CA, USA). RNA concentration, quality, and integrity were assessed with a nanodrop spectrophotometer (Thermo Scientific, Waltham, MA, USA). Approximately 3 μg of RNA was used for library preparation. For the sequencing, mRNA was purified from total RNA using poly-T oligo-attached magnetic beads. RNA fragmentation was achieved with divalent cations and Illumina proprietary buffer. First-strand cDNA was synthesized with random oligonucleotides and SuperScript II, followed by second-strand cDNA synthesis using DNA Polymerase I and RNase H. Overhangs were converted to blunt ends and enzymes were removed. DNA fragments were adenylated at the 3′ ends and Illumina PE adapter oligonucleotides were ligated for hybridization. To select 400–500 bp cDNA fragments, we purified the library using AMPure XP system (Beckman Coulter, Beverly, CA, USA). Fragments with adaptors on both ends were enriched with Illumina PCR Primer Cocktail in a 15-cycle PCR, purified (AMPure XP system), and quantified using an Agilent high-sensitivity DNA assay on a Bioanalyzer 2100 (Agilent, Technologies Inc, Santa Clara, CA, USA). The library was sequenced on the NovaSeq 6000 platform (Illumina) at Shanghai Personal Biotechnology Co., Ltd, Shanghai, China.

### 2.7. Transcriptome Data Analysis

The mRNA expression profiles of ten sequencing libraries were analyzed using RNA-seq. Raw FASTQ reads underwent quality control and filtering with fastp (v0.22.0) to remove low-quality sequences [31]. The filtered reads were aligned to the pig reference genome (Sscrofa 11.1) using HISAT2 (v2.1.0) [32]. The gene expression was quantified using HTSeq (v0.9.1) [33], and the read counts were normalized into FPKM. The differential expression genes (DEGs) were assessed with DESeq (v1.38.3) [34]. These DEGs were identified based on thresholds of |log2FoldChange| > 1 and *p*-value < 0.05. The bidirectional clustering of DEGs was performed using Pheatmap (v1.0.12) in R [35], and functional annotation and enrichment analyses were carried out using BioMart for gene name conversion [36]. We used the top GO (v2.50.0) for Gene Ontology (GO) enrichment analysis [37], and ClusterProfiler (v4.6.0) for KEGG pathway analysis [38]. Further, Gene Set Enrichment Analysis (GSEA) (v4.1.0) was used for assessing the gene set enrichment analysis [38]. Correlation analysis of DEG expression with meat quality traits and fatty acid composition was performed with the R package Hmisc [39], and results were visualized using Pheatmap R package [39]. Protein–protein interaction (PPI) networks were analyzed using STRING (v12.0) [40], and Cytoscape (v3.10.2) was employed to identify subnetworks of key genes from the PPI network [41].

### 2.8. Validation of DEGs

To verify the reliability of the RNA-seq data, RT-qPCR was performed on ten candidate genes (EIF4E, PRKAR2A, PRKAG2, FASN, MSTN, MYOG, INHBB, EGR2, PPARGC1A, and PPP1R3B). We used the four samples in each group with three replications per sample, and same aliquot of total RNA from these samples was utilized for RNA-seq detection for each gene. Primers were designed by NCBI primer blast and primer 5.0, and the PCR condition and proportion were the same as our previous work with each primer concentration of 10 mol/μL; the GAPDH gene was used as internal controls [42]. The different level of gene expression between two groups was tested by software GraphPad Prism (v8.0.2). The results were presented as mean and standard error. The nucleotide sequences of primers were listed in (Table S2).

### 2.9. Detection of Longissimus Dorsi Muscle Metabolites

Five LD muscle samples from each breed were taken for preparing the detection of metabolites, as suggested by [43]. Polar metabolites were analyzed using a UHPLC system (Vanquish, Thermo-Fisher Scientific) with a Waters ACQUITY UPLC BEH Amide column (2.1 mm × 50 mm, 1.7 μm) connected to an Orbitrap Exploris 120 mass spectrometer (Thermo). The mobile phase included 25 mmol/L ammonium acetate and 25 mmol/L ammonium hydroxide in water (pH 9.75) as phase A, and acetonitrile as phase B. The auto-sampler was kept at 4 °C, and 2 μL of sample was injected. The mass spectrometer was operated in information-dependent acquisition (IDA) mode, with control via Xcalibur software (v4.3). Electrospray ionization (ESI) parameters were as follows: sheath gas flow rate of 50 Arb, auxiliary gas flow rate of 15 Arb, capillary temperature of 320 °C, full MS resolution of 60,000, MS/MS resolution of 15,000, stepped normalized collision energy (SNCE) of 20/30/40, and spray voltages of 3.8 kV (positive mode) or −3.4 kV (negative mode).

### 2.10. Analysis of Longissimus Dorsi Muscle Metabolites

Untargeted metabolite analysis was performed using total ion chromatogram (TIC) data. The raw data for both positive and negative ion metabolite concentrations were processed with XCMS software (v4.3) [44], which managed peak alignment, retention time correction, and peak area extraction. Following data processing with XCMS, metabolite identification and data preprocessing were carried out [45]. Multivariate statistical analyses, including Principal Component Analysis (PCA), Partial Least Squares–Discriminant Analysis (PLS-DA), and Orthogonal Partial Least Squares–Discriminant Analysis (OPLS-DA), were conducted using R package Ropls [46]. Differential metabolite analysis was performed with a significance threshold of |Log2Fc| > 1 and an adjusted *p*-value < 0.05 [35]. Volcano plot and a cluster heat map were generated to visualize the results [47]. The KEGG (Kyoto Encyclopedia of Genes and Genomes) database was utilized to analyze the differentially expressed metabolic pathways [48].

### 2.11. Statistical Analyses

The fatty acid profiling, amino acids profiling, and meat quality traits data were statistically analyzed using SPSS version 26 (IBM Corp., Armonk, NY, USA). The descriptive statistics, including means and standard deviations (SD) of all samples, were calculated for each fatty acid, amino acid, and meat quality trait, and significance of each variable was analyzed using a Student *t*-test between both pig breeds. For all comparisons, a *p*-value < 0.05 was considered statistically significant. Furthermore, the correlation between some DEGs and top 30 DEMs (15 upregulated and 15 downregulated metabolites) was analyzed through Pearson correlation analysis method using Hmisc R package and visualized using Pheatmap [39,46].

## 3. Results

### 3.1. Comparion of Meat Quality Traits of LD Muscle Between SBP and LWLDP Breeds

A comparison of the meat quality traits of the LD muscle reveals significant differences in the Songliao Black Pig (SBP) and Large White × Landrace Pig (LWLDP) breeds (Table 1). The SBP exhibits notably higher back fat thickness (*p* < 0.001) with an average of 35.7 mm compared to 17.2 mm in the LWLDP. Similarly, the SBP demonstrates a higher meat color with an average score of 3.5 compared to 2 in the LWLDP. Marbling score was significantly (*p* < 0.001) lower in the LWLDP, having an average of 3.5 compared to 1.5 in the SBP. In terms of pH 24 h, the SBP has a slightly higher pH 24 h (*p* < 0.01) of 5.70, whereas the LWLDP has a value of 5.54. The drip loss at 48 h was significantly lower in the SBP at 2.70% compared to 4.01% in the LWLDP (*p* < 0.001). Shear force was notably lower in the SBP (*p* < 0.001), with an average force of 41.38 Newton (N) compared to 59.68 N in the LWLDP.

### 3.2. Fatty Acid and Animo Acid Profile of LD Muscle Between SBP and LWLDP Breeds

GC-MS analysis detected a total of thirty-seven different FAs (Table 2). Among them, sixteen showed significant differences. The SBP exhibited a lower total saturated fatty acid concentration (117.1 ± 29.5) compared to the LWLDP (175.32 ± 35.69, *p* < 0.05). Additionally, the SBP had significantly lower monounsaturated fatty acids (116.42 ± 25.4) than the LWLDP (168.08 ± 35.95, *p* < 0.05). In terms of polyunsaturated fatty acids, the SBP also showed reduced levels (36.11 ± 10.1) compared to the LWLDP (48.69 ± 5.05, *p* < 0.05). Notably, the SBP had higher docosahexaenoic acid (DHA) levels (0.86 ± 0.36) compared to the LWLDP (0.42 ± 0.06, *p* < 0.05), as detailed in Table S3. Further, we found no significant differences in amino acid profiles during a comparison of each amino acid between both breeds (Table 3), as detailed in Table S4.

### 3.3. Summary Statistics for RNA-Seq Data

Raw reads were obtained from ten cDNA libraries with average reads between 41,996,006 and 69,106,570 (Table S5). After trimming, a consistently high percentage of reads, ranging from 98.10% to 98.77%, and bases, ranging from 97.94% to 98.47%, were retained. The mapping efficiency to the pig reference genome (Sscrofa 11.1) was also notable, with 97.68% to 98.18% of reads successfully aligned. Within these alignments, a high proportion of reads, between 96.29% and 97.48%, mapped to gene regions, with 91.79% to 94.39% mapping specifically to exons.

### 3.4. Comparative Transcriptome Analysis of LD Muscle Between SBP and LWLDP Breeds

**Figure 1:**
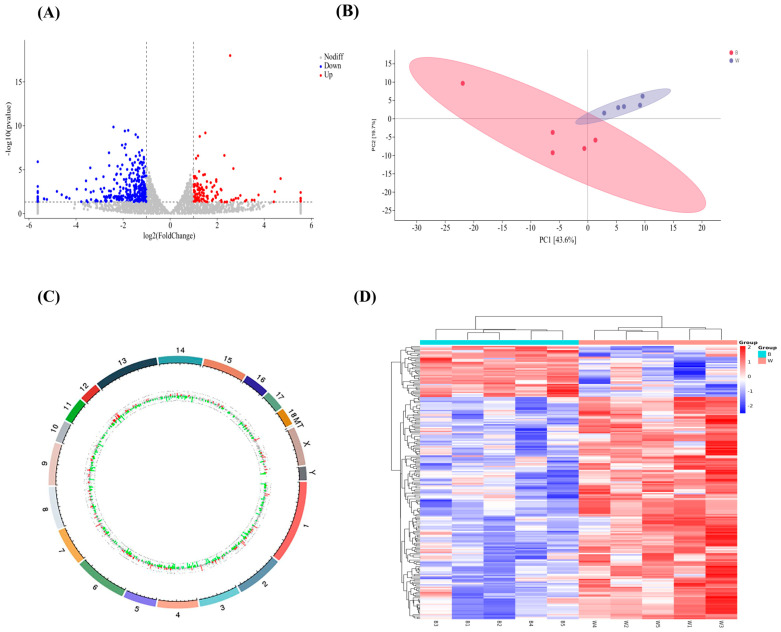
Comparative transcriptome analysis between SBP and LWLDP. (**A**) Volcano plot of DEGs with thresholds set at |log2FC| > 1 and *p*-value < 0.05 with downregulated genes in SBP are shown in blue, while upregulated genes in LWLDP are in red. (**B**) Principal component analysis (PCA) plot of expressed genes, with “B” representing SBP and “W” representing LWLDP, demonstrating the separation between the two breeds based on gene expression profiles. (**C**) Circular plot displaying the distribution of DEGs across different chromosomes. (**D**) Heatmap illustrating the expression levels of DEGs in each sample provides a visual comparison of gene expression patterns.

We identified a total of 16,531 expressed genes in the SBP and LWLDP (Table S6). Among these, 496 were classified as DEGs, with 362 genes downregulated and 134 upregulated in the LWLDP (Figure 1A, Table S7). The PCA plot showed a distinct separation among breed samples (Figure 1B). The expressed genes showed a widespread distribution across all chromosomes, with chromosomes 1, 2, 7, and 12 being more prominently represented (Figure 1C), while the distribution of differentially expressed genes within each sample was shown in (Figure 1D). Most genes had very low expression levels (0–0.01 FPKM), with averages of 7173.2 for the SBP and 7380.6 for the LWLDP. Moderate expression levels (1–10 FPKM) were observed, with averages of 5838.4 for the SBP and 5876.2 for the LWLDP. High expression levels (>1000 FPKM) were relatively rare, with averages of 41 for the SBP and 40 for the LWLDP. The top thirty highly expressed upregulated and downregulated genes based on the log2 FC value were shown in Table 4.

### 3.5. Functional Analysis of DEGs Involved in Meat Quality Traits and Types of Expressed Genes

**Figure 2:**
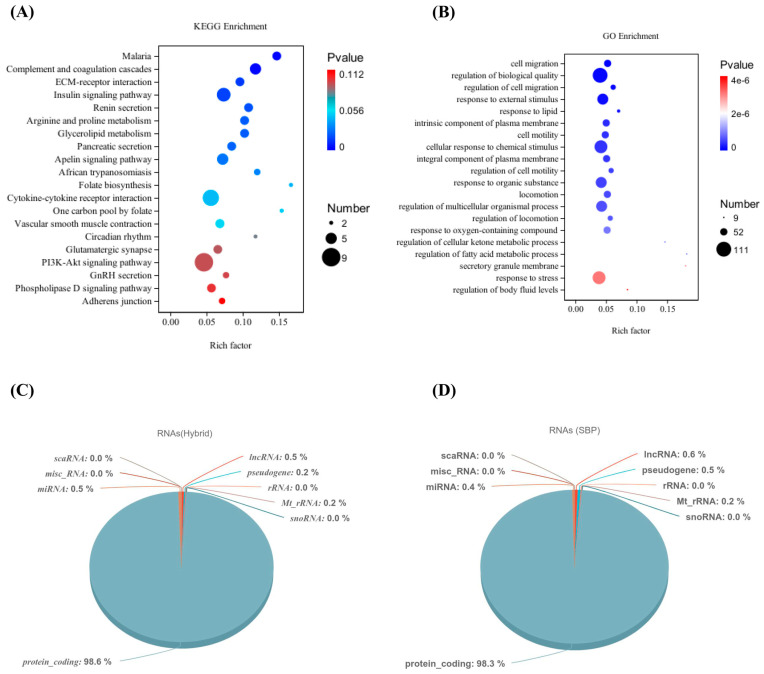
Functional analysis of DEGs and RNA types in SBP and LWLDP breeds. (**A**) The top 20 KEGG pathways for DEGs of both breeds. (**B**) The top 20 GO pathways. (**C**) The percentage and types of RNAs in SBP. (**D**) The percentage and types of RNAs in LWLDP breed.

Functional analysis found that several KEGG pathways, including insulin signaling, ECM–receptor interaction, glycerolipid metabolism, and PI3K-Akt pathways, are crucial for regulating meat quality. Genes of the insulin signaling pathway (PPP1R3B, PPARGC1A, SOCS1, EIF4E, PRKAR2A, PRKAG2, and FASN) are important regulators in balancing the fatty acid metabolism and catabolism. Other pathways such as the PI3K-Akt signaling pathway containing KIT, ANGPT4, THBS1, OSMR, SPP1, EIF4E, TNC, COL6A5, and FLT4 genes are potential contributors in fat deposition and meat quality. The top 20 KEGG pathways are shown in (Figure 2A) and detailed in (Table S8). GO pathways such as the cell migration pathway, involving CHGA, ANGPT4, KIT, and CXCR5 genes, contribute to the regulation of the biological quality and response to external stimuli, contain ASIC1, KCNJ8, and CHGA genes. Lipid metabolism, a potent contributor to fat deposition, involving KCNJ8 and RORC genes and pathways related to cell motility involving ANGPT4 and KIT genes are the most important in regulating meat quality. The top 20 GO pathways are mentioned in (Figure 2B) and detailed in (Table S9). According to gene annotation, protein-coding genes are the most prevalent, accounting for 98.34% and 98.61% of the expressed genes in the SBP and LWLDP, respectively. Long non-coding RNAs (lncRNAs) are the second most common, representing 0.59% and 0.47% of the expressed genes, followed by microRNAs (miRNAs) at 0.36% and 0.51%. Other RNA types, such as pseudogenes and mitochondrial tRNAs, are present but in lower percentages. Meanwhile, Y_RNA, vault RNA, and TRJ_gene are negligible or absent in both breeds (Figure 2C,D).

**Figure 3:**
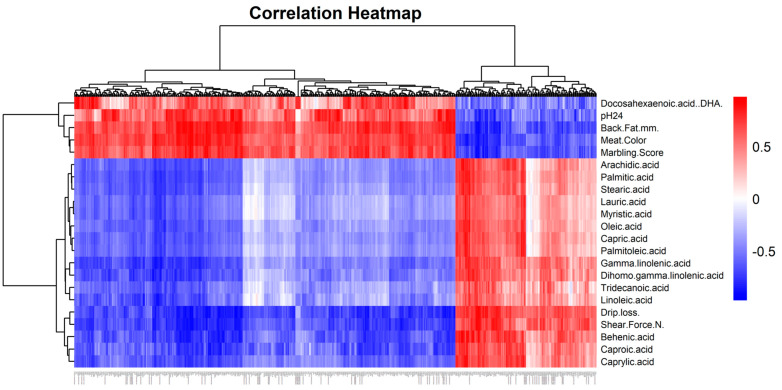
Correlation heat map showing the relationships between DEGs and fatty acids, as well as meat quality traits in SBP and LWLDP. Red color indicated a positive correlation and blue showed negative correlation.

**Figure 4:**
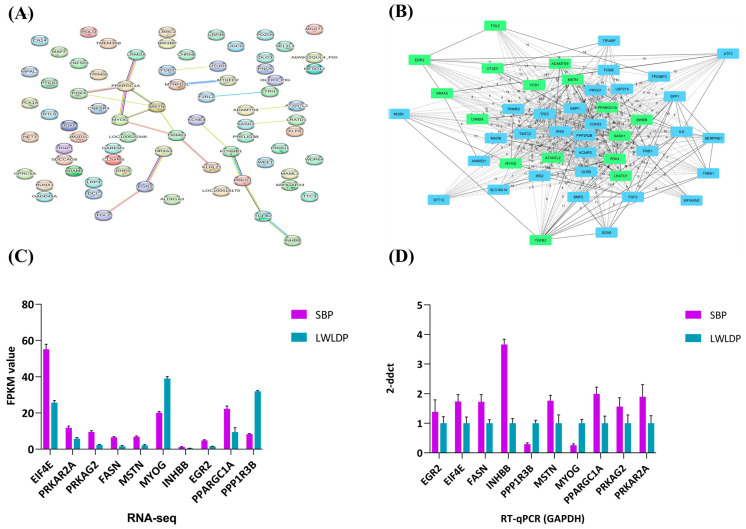
Functional analysis of meat quality genes and validation results by RT-qPCR. (**A**) PPI network plot showing highly correlated genes (r > 0.9, *p* < 0.001) associated with meat quality traits and fatty acids. (**B**) Subnetwork of key genes highlighting the primary interactions: green highlighting shows key genes and their interaction with other DEGs. (**C**) RNA-seq results of ten selected DEGs in SBP and LWLDP breeds. (**D**) Relative expression level verified by GAPDH (control) of the ten selected DEGs in SBP and LWLDP breeds.

A Pearson correlation analysis of DEGs with FAs and meat quality traits identified 437 genes that were significantly correlated (r > 0.7, *p* < 0.05) with FAs and meat quality traits (Figure 3 and Table S10). We selected the genes with a correlation threshold of (r > 0.9, *p* < 0.001), in which 75 genes were correlating with various traits such as backfat thickness (20 genes), drip loss (16 genes), marbling score (9 genes), meat color (6 genes), pH24 (13 genes), shear force (11 genes), and fatty acids in the longissimus dorsi muscle (27 genes). Of these, 37 genes were correlated with multiple traits, detailed in (Table S11). The PPI network of 75 highly correlated genes identified the functional interactions, in which 25 important genes such as Leucine-Rich Melanocyte Differentiation Associated (LRMDA), Pyruvate Dehydrogenase Kinase 4 (PDK4), Peroxisome Proliferator-Activated Receptor Gamma Coactivator 1 Alpha (PPARGC1A), Myostatin (MSTN), Myogenin (MYOG), Tripartite Motif Containing 63 (TRIM63), Muscle RING-Finger Protein-1 (MURF1), Kelch Like Family Member 21 (KLHL21), Nuclear Receptor Subfamily 4 Group A Member 3 (NR4A3), Early Growth Response 2 (EGR2), Fibroblast Growth Factor 2 (FGF2), YOD1 Deubiquitinase (YOD1), OTU Deubiquitinase 1 (OTUD1), Methylenetetrahydrofolate Dehydrogenase (NADP Dependent) 2 Like (MTHFD2L), Methylenetetrahydrofolate Dehydrogenase (NADP Dependent) 2 (MTHFD2), Coagulation Factor II (Thrombin) Receptor-Like 2 (F2RL2), Thyrotropin-Releasing Hormone (TRH), ADAM Metallopeptidase with Thrombospondin Type 1 Motif 9 (ADAMTS9), Ataxin 7 Like 2 (ATXN7L2), SAM And SH3 Domain Containing 1 (SASH1), Potassium Voltage-Gated Channel Subfamily E Regulatory Beta Subunit 4 (KCNE4), Potassium Calcium-Activated Channel Subfamily M Regulatory Beta Subunit 1 (KCNMB1), Protein Kinase, cGMP-Dependent, Type I (PPKG1), Transforming Growth Factor Beta 2 (TGFB2), Inhibin Subunit Beta B (INHBB), and Lecithin Retinol Acyltransferase Domain Containing 1 (LRATD1) are involved in various biological processes, with significant roles in metabolism, growth, and cellular regulation (Figure 4A,B). Furthermore, GO analysis of these genes identified six key genes (MSTN, MYOG, INHBB, EGR2, NR4A3, and PPARGC1A) as being involved in the top 10 GO pathways, such as energy homeostasis, fat cell differentiation, muscle cell proliferation, muscle organ development, the regulation of muscle tissue development, the regulation of tissue development, skeletal muscle cell differentiation, skeletal muscle organ development, and skeletal muscle tissue development. Among these, the MYOG gene was highly expressed in the LWLDP, while others were more prominently expressed in the SBP. These are the most important genes that regulate the meat quality in pig breeds.

### 3.6. Tests and Verification

To validate the RNA-seq data, ten genes (EIF4E, PRKAR2A, PRKAG2, FASN, MSTN, MYOG, INHBB, EGR2, PPARGC1A, and PPP1R3B) were randomly selected for confirmation using RT-qPCR. The RT-qPCR results exhibited similar expression trends to those observed in the RNA-seq analysis (Figure 4C,D). It confirms the accuracy and reliability of the RNA-seq data. This validation supports the precision and effectiveness of the transcriptomic analysis in identifying the key regulators of fat deposition and meat quality.

### 3.7. Differential Expressed Metabolites (DEMs) and Functional Analysis of LD Muscle Metabolites Between SBP and LWLDP Breeds

**Figure 5:**
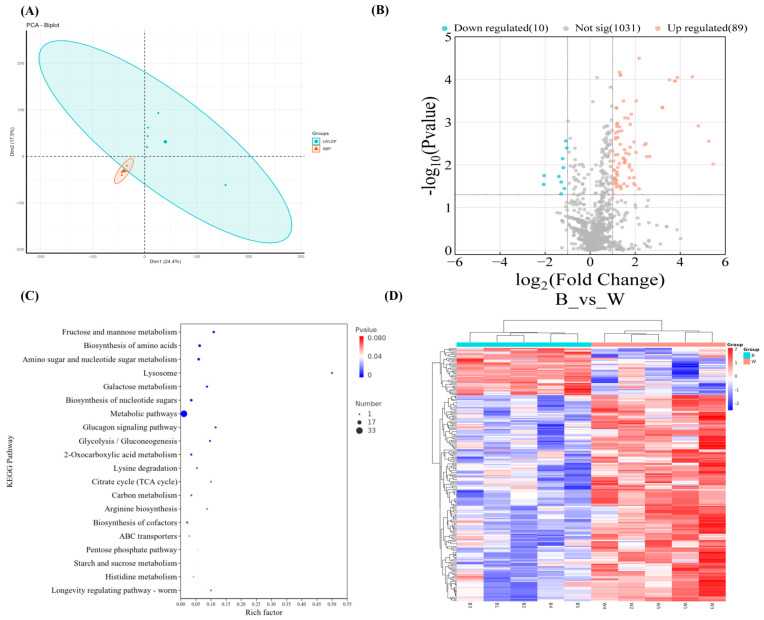
**The** DEMs analysis of LD muscle in SBP and LWLDP. (**A**) Principal component analysis (PCA) of LD muscles’ metabolites. (**B**) Volcano plot of differentially expressed metabolites. (**C**) Top 20 significantly enriched pathways associated with differential muscle metabolites. (**D**) Heatmap of differentially expressed metabolites across individual sample of SBP and LWLDP.

The untargeted metabolomics approach detected a total of 13,505 metabolites through ultra-high performance liquid chromatography (UHPLC), including 7188 negative and 6317 positive ion metabolites (Table S12). Multivariate analyses revealed distinct metabolite profiles between SBP and LWLDP pigs. A PCA plot showed the significant variation among breeds (Figure 5A), while PLS-DA and OPLS-DA identified 1130 common metabolites (Table S13). The DEM analysis found 10 downregulated metabolites in the SBP and 89 upregulated metabolites in the LWLDP (Figure 5B). The top 20 metabolites with the highest VIP scores and log2FC values are listed in (Table 5). The KEGG pathway analysis of differential metabolites identified 58 pathways (Table S14), with the top 20 shown in (Figure 5C). Key pathways include fructose and mannose metabolism, amino acid biosynthesis, nucleotide sugar metabolism, lysosome function, and glucagon signaling. Notably, the fructose and mannose metabolism pathway were upregulated in the LWLDP, with metabolites like mannose and fructose 6-phosphate. Similarly, galactose metabolism showed the upregulation of glucose 1-phosphate in the LWLDP. The biosynthesis of the amino acid pathway showed the upregulation of lysine in the LWLDP and the downregulation of the histidinol metabolite in the SBP; the amino sugar and nucleotide sugar metabolism pathway was notably active in the LWLDP, with the upregulation of metabolites like glucose 1-phosphate and mannose 6-phosphate. The cluster heat map shows the distribution of these differentially expressed metabolites across the samples (Figure 5D).

### 3.8. Pearson Correlation of Key Genes and Metabolites

**Figure 6:**
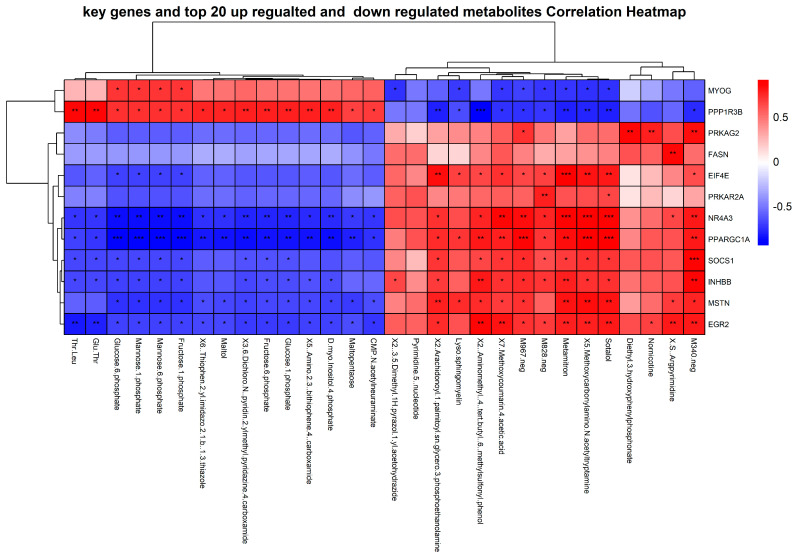
Correlation heatmap illustrating the associations between key genes and the top 30 most upregulated and downregulated metabolites in SBP and LWLDP. The clustering heatmap visually represents the strength and direction of these correlations. Red colors indicate a positive correlation, while blue colors signify a negative correlation. Statistical significance is indicated by asterisks: * = *p* < 0.05, ** = *p* < 0.01, *** = *p* < 0.001, highlighting the most significant associations between key genes and metabolites.

The Pearson correlation of key genes (MSTN, MYOG, INHBB, EGR2, NR4A3, and PPARGC1A), including the insulin signaling pathway genes (PPP1R3B, PPARGC1A, SOCS1, EIF4E, PRKAR2A, PRKAG2, and FASN), with the top 30 metabolites (15 upregulated and 15 downregulated) revealed significant relationships. Specifically, PPP1R3B showed a strong positive correlation |r > 0.8, *p* < 0.01| with metabolites such as Thr-Leu, Amino-2-3-bithiophene-4-carboxamide, Maltol, D-myo-Inositol-4-phosphate, Fructose-6-phosphate, Glucose-1-phosphate, and 3-6-Dichloro-N-pyridin-2-ylmethyl-pyridazine-4-carboxamide. The MYOG gene correlated with Mannose-6-phosphate, Fructose-1-phosphate, Mannose-1-phosphate, and Glucose-6-phosphate. Both these genes had a higher expression in the LWLDP breed. Conversely, NR4A3 and PPARGC1A showed a strong negative correlation with most upregulated metabolites, while FASN demonstrated weaker correlations. In contrast, most insulin signaling pathway genes and key genes exhibited significant positive correlations with downregulated metabolites (Figure 6).

## 4. Discussion

In the pig industry, meat quality is one of the most important economic traits globally, and it is influenced by multiple factors, including heredity and the environment [49]. We compared the meat quality traits, FA profile, AA profile, and transcriptomic and metabolomic profiles in the longissimus dorsi muscle of the Songliao Black Pig (SBP) and Large White × Landrace Pig (LWLDP) breeds. Our aim was to identify the molecular and metabolic differences contributing to breed-specific differences in meat quality traits, offering insights for improving pork quality.

It is well-known that indigenous pigs exhibit better meat quality compared to commercial lean pigs [11]. The SBP is the first lean meat female hybrid breed developed from the Jilin local sow, Duroc, and Landrace pigs, and exhibits superior meat quality traits. Compared to the LWLDP, the SBP has thicker backfat, higher marbling scores, better meat color, and higher pH at 24 h post-slaughter. In contrast, the LWLDP shows significantly higher shear force and drip loss. These findings align with previous studies on native breeds [50,51,52,53,54], Złotnicka Spotted Pigs exhibit greater backfat thickness than Złotnicka Spotted × Duroc hybrids. Similarly, the PBP shows higher backfat, intramuscular fat, and pH24 compared to the LWLDP [55]. Meat from native lean breeds tends to exhibit a higher quality, characterized by a slower decline in pH post-mortem. Rapid pH drops can increase the drip loss, reduce the water-holding capacity, and alter the meat color [56,57]. Additionally, the pH value is positively correlated with the glycogen content in muscle [58,59,60]. We hypothesize that the LD muscle of the SBP has lower glycogen levels and a slower rate of pH decline, leading to higher pH values in these muscles. The meat color is closely linked to the pH levels; typically, a lower pH is associated with a lighter meat color and a reduced water-holding capacity [61,62]. Previous research has shown that local Chinese pig breeds had higher-quality meat than commercial Western pigs, such as the Large White, a commercial breed with low meat quality and a quick growth rate [2,19,20]. In our study, we found obvious differences in meat quality between the SBP and commercial LWLDP.

Pork taste is largely influenced by its fatty acid composition; at the time of cooking, a lot of fatty acids react to produce volatile aromatic compounds. Meat flavor development is positively impacted by MUFAs, but PUFAs inhibit the Maillard reaction and thiamin degradation processes, which impacts the overall taste development [63,64,65]. PUFAs oxidize faster than SFAs, altering the taste and aroma while reducing the muscle juiciness and water-holding capacity [66,67]. Our findings show that the SBP has significantly lower fatty acid levels than the LWLDP, except for DHAs. This aligns with previous studies reporting a higher fatty acid content in commercial and native pig breeds [68]. Previous studies have demonstrated that adipose tissue is the largest metabolic energy reservoir, storing fat as triglycerides in adipocytes, depositing it as visceral, subcutaneous, intermuscular, and intramuscular fat, with the deposition intensity declining over time [69]. The unique metabolic characteristics of each fat deposit affect the body’s overall metabolism through the release of hormones, adipocytokines, and regulatory proteins [70]. These hormones affect various processes, including nutritional intake, inflammatory response, and insulin sensitivity [71]. Hormonal regulation differs among breeds and influences both fat accumulation and utilization processes [72]. The molecular mechanisms governing differences in fat accumulation and utilization remain unclear. Local pig breeds generally grow more slowly than commercial breeds, with distinct fatty acid deposition patterns.

A comparison of amino acid profiles showed no significant differences between the SBP and LWLDP. Similarly, minimal variations in threonine and isoleucine levels were observed between indigenous Prestice Black-Pied pigs and a commercial hybrid LWLDP [73]. Another study also found no significant differences in amino acid proportions between breeds [74]. These results are suggesting a little variation in amino acid composition between breeds. Our hypothesis is that, when pigs are reared in the same environment, the same diet may show less difference in the amino acid profiles of pig breeds.

A further comparison of muscle metabolites revealed significant differences between the SBP and LWLDP. The LWLDP exhibited a higher number of metabolites involved in fructose and mannose metabolism, amino acid biosynthesis, amino sugar and nucleotide sugar metabolism, glycogen signaling, gluconeogenesis, and other metabolic pathways. Similar patterns have been reported in previous studies comparing the metabolomic profiles of LD muscles in DLY and CB pig breeds [11]. We hypothesized that the higher muscle glycogen content accelerates the post-mortem pH decline through the increased lactic acid production during glycolysis. This rapid pH drop can impair meat quality, leading to pale, soft, and exudative (PSE) meat.

To investigate the superior meat quality of the SBP and higher fatty acid levels in the LWLDP, we analyzed the gene expression in the longissimus dorsi muscle. We identified 496 differentially expressed genes (DEGs), with 437 linked to meat quality traits. Key pathways include insulin signaling, PI3K-Akt, ECM–receptor interaction, and glycerolipid metabolism. Gene Ontology analysis highlighted muscle proliferation, fatty acid metabolism, and tissue development, providing insights into the genetic factors affecting meat quality, consistent with the findings in local pig breeds [2,3,75]. These DEGs likely drive the differences in meat quality traits; the PPI network highlighted 25 core genes, with GO enrichment analysis identifying 10 key pathways with genes like MSTN, MYOG, INHBB, EGR2, NR4A3, and PPARGC1A found to significantly impact the meat quality.

**Figure 7:**
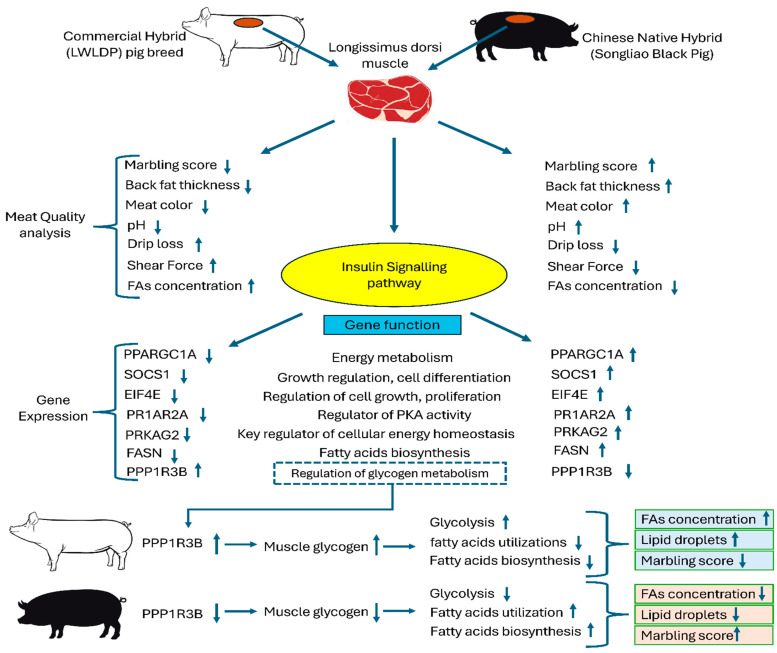
Insulin signaling pathways influencing meat quality and fat deposition patterns in SBP and LWLDP breeds. In this figure, the upward arrows, annotated with specific parameters (such as meat quality traits and genes), indicate increased levels in breeds, after comparing the Songliao Black Pig (SBP) to LWLDP pigs, while downward arrows indicate decreased levels. In SBP, higher expression of PPARGC1A, SOCS1, EIF4E, PRKAR2A, PRKAG2, and FASN genes is associated with enhanced energy metabolism, growth regulation, and cellular energy homeostasis. Conversely, PPP1R3B shows higher expression in LWLDP, indicating increased glycogen metabolism. SBP exhibits a higher marbling score with lower FA concentration, linked to elevated insulin signaling gene expression. In contrast, LWLDP shows higher FA concentration, possibly due to disrupted energy homeostasis and an imbalance between fat synthesis and catabolism, resulting in increased fatty acid concentrations, but decreases marbling scores, impacting overall meat quality.

Our transcriptome analysis showed a higher expression of insulin signaling genes (PPARGC1A, SOCS1, EIF4E, PRKAR2A, PRKAG2, and FASN) in the SBP, while PPP1R3B was more expressed in the LWLDP. Previous studies have also highlighted the importance of the insulin signaling pathway in fat deposition [23,76]. In addition, Fatty Acid Synthase (FASN) is an enzyme that catalyzes the synthesis of fatty acids from acetyl-CoA and malonyl-CoA precursors, and Peroxisome Proliferator-Activated Receptor Gamma Coactivator 1-Alpha (PPARGC1A) is a PGC-1α that is involved in the regulation of cellular energy metabolism, and is particularly involved in mitochondrial biogenesis and respiration; previous studies have mentioned that these genes are involved in fat metabolism and energy regulation [77,78]. While PPP1R3B shows a high expression in the LWLDP breed while other genes related to fat metabolism (like PPARGC1A, SOCS1, EIF4E, PRKAR2A, PRKAG2, and FASN) show a low expression, it indicates a unique metabolic regulation in the SBP. A correlation analysis of PPP1R3B and metabolites showed a highly significant correlation with Mannose-6-phosphate, Fructose-1-phosphate, Mannose-1-phosphate, and Glucose-6-phosphate. A higher PPP1R3B expression might enhance glycogen storage, which could divert glucose utilization towards fat synthesis, leading to higher fatty acid concentrations, while other genes that are related to energy metabolism, cellular energy homeostasis, and fatty acids synthesis show a very low expression; possibly, stored glycogen at a higher level did not utilize the fat, while other studies mention that the deposition of fat in animals represents the balance between fat synthesis and catabolism. Once the original balance is disrupted, fat deposition increases or body fat decreases, thereby affecting the meat quality of animals [79]. Our study concluded the molecular mechanisms and genes that regulate this process. In our hypothesis, a higher deposition and utilization of glycogen and less fat consumption disturb the fat deposition and catabolism, resulting in a decrease in the meat quality of the LWLDP breed. However, this might not improve the marbling quality since intramuscular fat deposition (linked to marbling) is primarily regulated by FASN and PPARGC1A [80]. Thus, the LWLDP breed might have a higher overall lipid content, but not better meat marbling. This unique molecular mechanism of balancing fat synthesis and catabolism can be utilized to improve meat quality (Figure 7).

In our study, the correlation of DEGs with meat quality traits and fatty acids found 25 key genes LRMDA, PDK4, PPARGC1A, MSTN, MYOG, TRIM63, KLHL21, NR4A3, EGR2, FGF2, YOD1, OTUD1, MTHFD2L, MTHFD2, F2RL2, TRH, ADAMTS9, ATXN7L2, SASH1, KCNE4, KCNMB1, PPKG1, TGFB2, INHBB, and LRATD1 genes have a potential role in meat quality. MYOG, encoding myogenin, regulates skeletal muscle development and interacts with genes like IGF-I, which stimulates myogenesis and correlates with the live and carcass weight in sheep by inducing MYOG expression [81]. This interaction highlights a complex regulatory network where MYOG collaborates with growth factors to regulate muscle development. Similarly, the myostatin (MSTN) gene, or Growth Differentiation Factor 8 (GDF8), negatively regulates muscle mass by inhibiting the proliferation and differentiation of myogenic cells, thus controlling skeletal muscle growth [82]. Previous studies have shown that MSTN mutations or the loss of function can increase muscle mass, reduce fat accumulation, and result in higher lean meat yield, improved tenderness (by reducing shear force), and enhanced red meat color [82,83]. The INHBB gene, encoding the beta B subunit of inhibin, is a potential candidate gene for meat quality traits in cattle. As a member of the TGF-beta superfamily, INHBB regulates physiological processes like cell proliferation and differentiation [84]; INHBB is associated with two key traits: intramuscular fat content (IFC) and meat color. IFC is crucial for meat quality, impacting the flavor, juiciness, and tenderness. INHBB likely influences the IFC through its role in lipid metabolism, with genetic variations affecting the expression of genes involved in lipid accumulation and intramuscular fat deposition [85,86]. Early Growth Response 2 (EGR2), also known as Krox20, is a transcription factor in the EGR gene family that regulates cell proliferation and differentiation. It influences meat quality by affecting muscle fiber development, myogenesis, intramuscular fat, meat color, and connective tissue formation [87]. The type and proportion of muscle fibers significantly affect the meat tenderness, color, and water-holding capacity. A higher proportion of oxidative muscle fibers (type I) is linked to improved tenderness and juiciness. NR4A3, a gene encoding a member of the NR4A subfamily of nuclear receptors, regulates physiological processes such as cell proliferation, differentiation, and metabolism [88]. In broiler chickens, muscle quality issues like myo-degeneration, fibrosis, and altered muscle fibers are linked to metabolic imbalances. A dysregulated NR4A3 may exacerbate these conditions by upregulating ENO3, increasing glycolytic activity, and contributing to disorders like wooden breast disease [89]. Peroxisome proliferator-activated receptor gamma coactivator 1-alpha (PPARGC1A) plays a key role in meat quality by promoting the development of slow-twitch oxidative (type I) muscle fibers. This influences the meat tenderness, color, fatty acid composition, oxidative capacity, pH, and water-holding capacity [81]. The Leucine Rich Motif Containing 1 (LRMDA) gene can influence the proportion of different muscle fiber types (Type I vs. Type II), and IMF in muscles, which, in turn, affects the overall quality of pork [75]. The PDK4 gene encodes a mitochondrial protein that inhibits the pyruvate dehydrogenase complex, influencing lipid metabolism. Its expression promotes fatty acid oxidation and reduced glucose utilization, supporting intramuscular fat (IMF) deposition, which enhances meat flavor and tenderness [75]. TRIM63 gene is also well-known for its meat quality traits in pigs. Its involvement in muscle atrophy and hypertrophy, along with its regulatory effects on muscle fiber composition and fat deposition, makes it a vital target for genetic studies aimed at improving pork quality [75]. The regulation of fat deposition, particularly IMF, is essential for enhancing meat quality. Higher IMF levels are associated with improved flavor and tenderness in pork. KLHL21 may play a role in lipid metabolism pathways that affect how fat is stored within muscle tissues. Studies have shown that genes involved in fat metabolism significantly influence IMF levels, which are key determinants of meat quality [90]. These genes play important roles in cell proliferation, energy metabolism regulation, and the development of muscle and fat. This study explains the reasons for the differences in meat quality traits and flavor substances between the two pig strains. It provides a theoretical basis for in-depth research on the Songliao Black Pig and the utilization of Chinese local pigs to obtain high-quality meat pig strains and new breeds.

## 5. Conclusions

This study successfully identified the differences in meat quality traits, fatty acid (FA) profiles, amino acid (AA) profiles, gene expression, and metabolic pathways that regulate the meat quality between Songliao Black Pig (SBP) and Large White × Landrace Pig (LWLDP) breeds. Specifically, the SBP exhibited significantly lower levels of SFA and UFA, along with higher concentrations of docosahexaenoic acid (DHA). Lower levels of SFA and UFA are associated with an improved fat composition, contributing to a more desirable meat texture, enhanced flavor, and a reduced risk of lipid oxidation. Conversely, the higher DHA levels observed in SBP meat enhance its nutritional profile, as DHA is well-known for its anti-inflammatory and health-promoting benefits. These factors could significantly improve consumer choice and preference for SBP meat. The study also identified 25 key genes involved in regulating meat quality. Particularly, genes of the insulin signaling pathway play a crucial role in fat deposition and metabolic regulation. Furthermore, the gene–metabolite association analysis revealed distinct roles for PPP1R3B, MYOG, NR4A3, and PPARGC1A in regulating metabolite profiles, providing deeper insights into their potential functions. In conclusion, this study identified functional genes and elucidated the mechanisms associated with meat quality traits and gene–metabolite interactions involved in energy metabolism, muscle development, and fat deposition. These results provide valuable insights into the molecular mechanisms that regulate the meat quality between pig breeds along with local pigs to obtain high-quality meat pig strains. This can be utilized for new breed development.

## Figures and Tables

**Table 1:** Comparative analysis of meat quality of longissimus dorsi muscle of SBP and LWLDP breeds.

Meat Quality Traits	SBP	LWLDP	Significance Level
	Mean ± SD	Mean ± SD	
Back Fat (mm)	35.70 ± 1.81	17.2 ± 3.26	***
Meat Color (score)	3.50 ± 0.27	20 ± 0.27	***
Marbling (score)	3.50 ± 0.44	1.50 ± 0.27	***
pH24 h	5.70 ± 0.07	5.54 ± 0.05	**
Drip Loss %	2.70 ± 0.12	4.01 ± 0.13	***
Shear Force (N)	41.38 ± 1.57	59.68 ± 5.42	***

Note: Mean represents the average of pig samples for each breed, while SD denotes the standard deviation. The significance of *p*-values is denoted as *** indicating *p* < 0.001, and ** indicating *p* < 0.01 denoting the level of significance in statistical analyses.

**Table 2:** Profile of fatty acid concentration (mg/100 g) in LD muscles of SBP and LWLDP breeds.

Fatty Acids	SBP	LWLDP	Significance Level
	Mean ± SD	Mean ± SD	
Butyric acid (C4:0)	0.11 ± 0.05	0.10 ± 0.02	ns
Caproic acid (C6:0)	0.01 ± 0.003	0.02 ± 0.004	**
Caprylic acid (C8:0)	0.04 ± 0.009	0.08 ± 0.02	**
Capric acid (C10:0)	0.48 ± 0.17	0.94 ± 0.28	**
Undecanoic acid (C11:0)	0.01 ± 0.003	0.02 ± 0.01	ns
Lauric acid (C12:0)	0.40 ± 0.22	0.74 ± 0.22	*
Tridecanoic acid (C13:0)	0.004 ± 0.002	0.007 ± 0.002	*
Myristic acid (C14:0)	6.26 ± 3.23	10.96 ± 3.05	*
Myristoleic acid (C14:1n5)	0.13 ± 0.06	0.23 ± 0.10	ns
Pentadecanoic acid (C15:0)	0.12 ± 0.06	0.19 ± 0.04	ns
Palmitic acid (C16:0)	67.40 ± 15.28	101.10 ± 20.20	**
Palmitoleic acid (C16:1n7)	13.70 ± 4.47	23.59 ± 7.12	*
Margaric acid (C17:0)	0.74 ± 0.41	1.11 ± 0.21	ns
Heptadecenoic acid (C17:1n7)	0.62 ± 0.33	0.99 ± 0.24	ns
Stearic acid (C18:0)	40.34 ± 10.09	57.90 ± 11.72	*
Elaidic acid (C18:1n9t)	0.73 ± 0.56	1.03 ± 0.26	ns
Oleic acid (C18:1n9c)	94.60 ± 17.27	132.20 ± 26.25	*
Linolelaidic acid (C18:2n6t)	0.02 ± 0.01	0.03 ± 0.005	ns
Linoleic acid (C18:2n6c)	28.70 ± 8.90	39.87 ± 4.38	*
Arachidic acid (C20:0)	1.08 ± 0.30	1.80 ± 0.51	*
γ-linolenic acid (C18:3n6)	0.16 ± 0.05	0.27 ± 0.04	**
Gadoleic acid (C20:1)	4.63 ± 2.20	7.00 ± 2.12	ns
α-linolenic acid (C18:3n3)	1.37 ± 0.74	1.84 ± 0.27	ns
Heneicosanoic acid (C21:0)	0.008 ± 0.003	0.01 ± 0.004	ns
Eicosadienoic acid (C20:2)	1.95 ± 1.05	2.90 ± 0.52	ns
Behenic acid (C22:0)	0.05 ± 0.007	0.10 ± 0.01	***
Dihomo-γ-linolenic acid (C20:3n6)	0.63 ± 0.16	0.96 ± 0.15	**
Eicosatrienoic acid (C20:3n3)	0.22 ± 0.16	0.39 ± 0.09	ns
Arachidonic acid (C20:4n6)	3.85 ± 0.64	4.72 ± 0.75	ns
Docosadienoic acid (C22:2n6)	0.15 ± 0.10	0.07 ± 0.03	ns
Lignoceric acid (C24:0)	0.03 ± 0.01	0.04 ± 0.008	ns
Eicosapentaenoic acid (C20:5n3)	0.07 ± 0.03	0.07 ± 0.003	ns
DHA (C22:6n3)	0.86 ± 0.36	0.42 ± 0.06	*
n3 PUFA	0.64 ± 0.25	0.68 ± 0.09	ns
n6 PUFA	1.20 ± 0.17	1.50 ± 0.22	*
n3/n6 PUFA	0.92 ± 0.17	1.09 ± 0.11	ns
SFA	117.10 ± 29.50	175.32 ± 35.69	*
UFA	152.50 ± 35.40	216.78 ± 38.83	*
MUFA	116.42 ± 25.40	168.08 ± 35.95	*
PUFA	36.11 ± 10.10	48.69 ± 5.05	*
T. FA	269.69 ± 64.60	392.10 ± 73.78	*

Note: Mean values represent the average fatty acid concentrations in pig samples within each breed, with standard deviation (SD) indicating variability. The significance of *p*-values is denoted as *** for *p* < 0.001, ** for *p* < 0.01, * for *p* < 0.05, and ns for *p* > 0.05 (no significance), reflecting the statistical significance level in the analysis. Abbreviations of corresponding fatty acids include the following: n3 PUFA (omega-3 polyunsaturated fatty acid) such C18:3n3, C20:5n3, and C22:6n3; n6 PUFA (omega-6 polyunsaturated fatty acid) including C18:2n6c, C18:3n6, C20:4n6, and C20:3n6; SFA (saturated fatty acid) such as C4:0, C6:0, C8:0, C10:0, C11:0, C12:0, C13:0, C14:0, C15:0, C16:0, C17:0, C18:0, C20:0, C22:0, and C24:0; MUFA (monounsaturated fatty acid) including C14:1n5, C16:1n7, C17:1n7, and C18:1n9c, and C20:1; PUFA (polyunsaturated fatty acids) encompassing all n3 and n6 PUFAs; and TFA (total fatty acids), representing the sum of all measured fatty acids, both saturated and unsaturated.

**Table 3:** Profile of amino acid concentration (µg/g) in longissimus dorsi muscle of SBP and LWLDP breeds.

Amino Acids	SBP	LWLDP	Significance Level
	Mean ± SD	Mean ± SD	
Methionine	43.66 ± 5.64	43.24 ± 8.43	ns
Threonine	14.63 ± 1.75	14.24 ± 2.01	ns
Serine	13.96 ± 1.33	14.08 ± 2.07	ns
Glutamic acid (Glutamate)	39.77 ± 5.41	44.13 ± 6.42	ns
Glycine	8.98 ± 1.13	8.63 ± 1.28	ns
Alanine	14.93 ± 2.19	14.20 ± 2.14	ns
Valine	16.16 ± 1.63	14.75 ± 2.42	ns
Methionine	7.34 ± 1.50	7.17 ± 1.05	ns
Isoleucine	11.61 ± 1.71	11.05 ± 1.46	ns
Leucine	20.16 ± 2.96	19.33 ± 2.55	ns
Tyrosine	7.66 ± 0.31	6.86 ± 1.27	ns
Phenylalanine	2.92 ± 0.32	3.07 ± 1.01	ns
Histidine	4.22 ± 0.62	3.72 ± 1.08	ns
Lysine	19.87 ± 3.24	19.27 ± 4.39	ns
Ammonia (NH4+)	0.39 ± 0.02	0.36 ± 0.06	ns
Arginine	7.46 ± 1.03	6.57 ± 1.09	ns
Proline	24.84 ± 2.70	24.79 ± 3.62	ns
Total amount (µg/g)	244.27 ± 28.02	237.50 ± 25.20	ns

Note: Mean values represent the average fatty acid concentrations in pig samples within each breed, with SD referring to the standard deviation, indicating the variability within the samples. The significance level is expressed through *p*-values, with “ns” denoting *p* > 0.05, indicating no statistical significance in the analyses.

**Table 4:** Top 30 highly upregulated and downregulated genes in LD muscles of SBP and LWLDP pig breeds.

Gene ID	Gene Name	Description	Log2FC Value	*p*-Value
ENSSSCG00000026686	PDZD9	PDZ domain containing 9	−2.40	1.47 × 10^−10^
ENSSSCG00000004071	CNKSR3	CNKSR family member 3	−1.77	3.56 × 10^−10^
ENSSSCG00000015334	PDK4	Pyruvate dehydrogenase kinase 4	−1.91	4.18 × 10^−10^
ENSSSCG00000011496	ADAMTS9	ADAM metallopeptidase with Yhrombospondin type 1 motif 9	−1.45	1.08 × 10^−9^
ENSSSCG00000023105	NET1	Neuroepithelial cell transforming 1	−1.36	2.13 × 10^−9^
ENSSSCG00000005385	NR4A3	Nuclear receptor subfamily 4 group A member 3	−1.88	1.37 × 10^−8^
ENSSSCG00000015579	PTGS2	Prostaglandin-endoperoxide synthase 2	−2.03	1.70 × 10^−8^
ENSSSCG00000003079	Un known	PVR cell adhesion molecule	−1.92	3.50 × 10^−8^
ENSSSCG00000035212	KLF6	Kruppel like factor 6	−1.18	6.64 × 10^−8^
ENSSSCG00000009432	DGKD	Diacylglycerol kinase delta	−1.31	1.13 × 10^−7^
ENSSSCG00000026043	Un known	Transglutaminase 3	−2.59	1.26 × 10^−7^
ENSSSCG00000003379	KLHL21	Kelch like family member 21	−1.86	2.25 × 10^−7^
ENSSSCG00000015808	ADAM9	ADAM metallopeptidase domain 9	−1.28	2.49 × 10^−7^
ENSSSCG00000006216	TRIM55	Tripartite motif containing 55	−1.45	3.03 × 10^−7^
ENSSSCG00000030165	MAFF	MAF bZIP transcription factor F	−1.10	3.96 × 10^−7^
ENSSSCG00000012773	PNCK	Pregnancy-upregulated nonubiquitous CaM kinase	2.55	1.10 × 10^−18^
ENSSSCG00000022797	PPP1R3B	Protein phosphatase 1 regulatory subunit 3B	1.50	6.85 × 10^−10^
ENSSSCG00000027875	CA14	Carbonic anhydrase 14	1.26	1.74 × 10^−9^
ENSSSCG00000000216	ASIC1	Acid sensing ion channel subunit 1	2.31	2.50 × 10^−7^
ENSSSCG00000010322	ZNF503	Zinc finger protein 503	1.19	2.77 × 10^−7^
ENSSSCG00000021059	ADORA1	Adenosine A1 receptor	1.11	5.44 × 10^−7^
ENSSSCG00000002456	CHGA	Chromogranin A	2.69	7.82 × 10^−6^
ENSSSCG00000023215	MAOB	Monoamine oxidase B	1.23	1.64 × 10^−5^
ENSSSCG00000031356	HES1	hes family bHLH transcription factor 1	1.48	3.65 × 10^−5^
ENSSSCG00000015984	HOXD4	Homeobox D4	1.41	4.18 × 10^−5^
ENSSSCG00000021638	NEU3	Neuraminidase 3	1.02	5.89 × 10^−5^
ENSSSCG00000006828	ATXN7L2	Ataxin 7 like 2	1.07	6.22 × 10^−5^
ENSSSCG00000024837	SYT12	Synaptotagmin 12	1.03	9.49 × 10^−5^
ENSSSCG00000012295	MAGIX	MAGI family member, X-linked	1.13	1.01 × 10^−4^
ENSSSCG00000000584	SLCO1A2	Solute carrier organic anion Transporter family member 1A2	4.70	1.08 × 10^−4^

**Table 5:** Statistics of the top 20 most significantly upregulated and downregulated metabolites in LD muscles of SBP and LWLDP breeds.

Metabolites Name	Log2FC	Significance Level	VIP
Diethyl 3-hydroxyphenyl phosphonate	−2.06	*	1.71
2-(3,5-Dimethyl-1H-pyrazol-1-yl) acetohydrazide	−2.04	**	1.78
2-(Amino methyl)-4-(tert-butyl)-((methyl sulfonyl) phenol	−1.39	*	1.82
7-Methoxycoumarin-4-acetic acid	−1.28	*	1.55
3-[4-[Acetyl(hydroxy)amino] butyl carbamoyl]-5-[3[acetyl(hydroxy)amino] propyl amino]-3-hydroxy-5-oxo pentatonic acid	−1.28	*	1.77
5-Methoxycarbonylamino-N-acetyl tryptamine	−1.22	*	1.99
(S)-Argpyrimidine	−1.19	**	1.70
Nornicotine	−1.14	*	1.63
2-Arachidonoyl-1-palmitoyl-sn-glycero-3-phosphoethanolamine	−1.07	**	1.94
Sotalol	−1.03	**	2.05
6-(Thiophen-2-yl) imidazo [2,1-b] [1,3] thiazole	5.46	**	1.84
Thr-Leu	5.26	**	2.02
5′-Amino-2,3′-bithiophene-4′-carboxamide	4.80	**	1.99
Maltol	4.53	***	2.09
D-myo-Inositol-4-phosphate	3.87	***	2.08
Fructose 6-phosphate	3.76	***	2.08
Glucose 1-phosphate	3.76	***	2.08
3,6-Dichloro-N-(pyridin-2-ylmethyl) pyridazine-4-carboxamide	3.52	***	2.08
Mannose 6-phosphate	3.20	***	2.08
Fructose 1-phosphate	3.20	***	2.08

Note: Variable importance in projection (VIP) assesses the contribution of each variable to the overall model’s predictive power. Log2 fold change (log2FC) quantifies the difference in expression levels between the breeds, indicating the magnitude of change. *p*-values denote the statistical significance of the differences observed, with *** indicating *p* < 0.001, ** indicating *p* < 0.01, and * indicating *p* < 0.05, reflecting the level of confidence in the results.

## Data Availability

The datasets generated or analyzed during the current study are available in the NCBI SRA under BioProject ID: PRJNA1176353.

## Supplementary Materials

The following supporting information can be downloaded at: https://www.mdpi.com/article/10.3390/ani14243560/s1, Table S1. Basic feed composition and nutritional levels of weaned piglets. Table S2. q-RT-PCR primers used for validation. Table S3. Profile of fatty acids concentration(mg/100 g) longissimus muscles of Songliao Black Pig (SBP) and commercial LWLDP pig breeds. Table S4. Profile of Amino acid concentration(µg/mg) in Longissimus dorsi muscles of Songliao Black Pig (SBP) and commercial LWLDP pig breeds. Table S5. Statistics summary of RNA-Seq data of longissimus dorsi muscle. Table S6. Normalized result of totally expressed genes read count to FPKM values. Table S7. Profile of express genes with DEGs in Songliao Black Pig (SBP) and Commercial LWLDP pig breeds. Table S8. Top 30 GO enrichment analysis. Table S9. Top 20 KEGG pathways of differential gene expression of SBP and LWLDP. Table S10. Pearson correlation of DEGs with meat quality traits and fatty acids. Table S11. Highly correlated 75 genes with fatty acids and meat quality traits. Table S12. Untargeted metabolites detected in LCMS/MS of Songliao Black Pig (SBP) and LWLDP pig breeds. Table S13. Common metabolites expressed in longissimus dorsi muscle of Songliao Black Pig (SBP) and Commercial LWLDP pig breeds. Table S14. The KEGG pathways of differential metabolites expressed in longissimus dorsi muscle of Songliao Black Pig (SBP) and commercial (LWLDP) pig breeds.
