# Supplementary material for: Transcriptome and Metabolome Insights into Key Genes Regulating Fat Deposition and Meat Quality in Pig Breeds
Source: Animals (Basel). 2024 Dec 10;14(24):3560. doi: 10.3390/ani14243560 (PMC11672692; doi:10.3390/ani14243560)
Supplement: Supplementary file 1 [file animals-14-03560-s001.zip › Table S1.pdf]

Table S1. Basic feed composition and nutritional levels of weaned piglets.

**Ingredients %**	**Weaned piglets**
Corn (Grade 1)	
Corn (Grade 2)	43.07
Puffed corn	
Barley	3.6
Wheat mixture	10
Rice bran	6
Soybean oil	2.5
Whey powder (low protein)	
Soybean meal (≥ 43%)	18.6
Soybean meal (≥ 45%)	
Soybean Enzymatic Hydrolyzed Protein (EP30)	
Expanded soybean	
Fish meal Japanese grade	
DDGS High Fat Level 1	4
Rice bran meal	8.5
4.44% Conservation Premix	
3.73% Premix for lactating sows	3.73
Total	100
Nutrient content	
Crude protein	≥17.0
Crude fiber	≤5.0
Coarse ash content	≤9.0
Calcium	0.3-1.3
Sodium chloride	0.2-1.5
Lysine	≥1.35
Water content	≤14.5
